# Plasma Transthyretin as a Predictor of Amnestic Mild Cognitive Impairment Conversion to Dementia

**DOI:** 10.1038/s41598-019-55318-0

**Published:** 2019-12-10

**Authors:** Yi-Ting Tien, Wei-Ju Lee, Yi-Chu Liao, Wen-Fu Wang, Kai-Ming Jhang, Shuu-Jiun Wang, Jong-Ling Fuh

**Affiliations:** 1 Section of Neurology, Internal Medicine Department, Chiayi Branch, Taichung Veterans General Hospital, Chiayi, Taiwan; 2 Neurological Institute, Taichung Veterans General Hospital, Taichung, Taiwan; 3 Faculty of Medicine, National Yang-Ming University Schools of Medicine, Taipei, Taiwan; 4 Dementia and Parkinson’s Disease Integrated Center, Taichung Veterans General Hospital, Taichung, Taiwan; 5 Center for Geriatrics and Gerontology, Taichung Veterans General Hospital, Taichung, Taiwan; 6 Brain Research Center, National Yang-Ming University, Taipei, Taiwan; 7 Division of Peripheral Nervous System Disorders, Taipei Veterans General Hospital, Taipei, Taiwan; 8 Division of General Neurology, Neurological Institute, Taipei Veterans General Hospital, Taipei, Taiwan; 9 Department of Neurology, Changhua Christian Hospital, Changhua, Taiwan; 10 Department of Holistic Wellness, Ming Dao University, Changhua, Taiwan

**Keywords:** Cognitive ageing, Neurophysiology, Biomarkers

## Abstract

Amnestic mild cognitive impairment (MCI) is a prodromal stage of dementia, with a higher incidence of these patients progressing to Alzheimer’s disease (AD) than normal aging people. A biomarker for the early detection and prediction for this progression is important. We recruited MCI subjects in three teaching hospitals and conducted longitudinal follow-up for 5 years at one-year intervals. Cognitively healthy controls were recruited for comparisom at baseline. Plasma transthyretin (TTR) levels were measured by ELISA. Survival analysis with time to AD conversion as an outcome variable was calculated with the multivariable Cox proportional hazards models using TTR as a continuous variable with adjustment for other covariates and bootstrapping resampling analysis. In total, 184 MCI subjects and 40 sex- and age-matched controls were recruited at baseline. At baseline, MCI patients had higher TTR levels compared with the control group. During the longitudinal follow-ups, 135 MCI patients (73.4%) completed follow-up at least once. The TTR level was an independent predictor for MCI conversion to AD when using TTR as a continuous variable (p = 0.023, 95% CI 1.001–1.007). In addition, in MCI converters, the TTR level at the point when they converted to AD was significantly lower than that at baseline (328.6 ± 66.5 vs. 381.9 ± 77.6 ug/ml, p < 0.001). Our study demonstrates the temporal relationship between the plasma TTR level and the conversion from MCI to AD.

## Introduction

Dementia due to Alzheimer’s disease (AD) is now the most common neurodegenerative disease in the 21^st^ century, and as this population rapidly grows, there will be an increasingly large economic burden^1^. Amnestic mild cognitive impairment (MCI) is the prodromal stage of AD, and the patients with MCI are distinct from normal aging people and progress to AD at the high rate of 10% to 15% per year^2,3^. In the clinical context, identifying patients with MCI who will develop AD within a particular period of time is important. Many studies have demonstrated potential biomarkers for AD conversion from prodromal stages, including amyloid-beta (Aβ) and tau proteins in cerebrospinal fluid (CSF), structural and functional neuroimaging features, and neuropsychological deficits in specific cognitive domains^4–7^. However, the correlations between these biomarkers and the rate of cognitive decline are not all the same. Looking for other blood-based biomarkers associated with the cognitive decline in MCI patients is critical for early diagnosis and early treatment in the future.

Transthyretin (TTR) is a compound mainly found in the epithelial cells of the choroid plexus in the brain, which carries the thyroid hormone thyroxine and retinol-binding protein bound to retinol^8,9^, and is also secreted by the liver into the blood. It can be detected both in plasma and CSF^9–11^. Additionally, TTR has also been considered a neuronal product in hippocampus and cerebral cortex^12,13^ as well as ligand that interacts with Aβ and disrupts Aβ fibrils^14^. Some studies have identified TTR as a candidate plasma biomarker for helping the diagnosis of, reflecting the disease severity of, and assessing the progression in AD^15–17^. Plasma TTR levels have been shown to be decreased in both MCI and AD patients compared to healthy controls, but no significant differences were detected between the two patient groups^16,17^. In AD patients, plasma TTR levels were significantly lower in AD cases with rapid cognitive decline and with severe cognitive impairment than in those with less rapid decline and less severe impairments^15^. However, no study has examined the longitudinal association between TTR levels and MCI-to-AD conversion.

In this study, we investigated the correlation between plasma TTR levels and the trend in the incidence of AD conversion from MCI subjects. We collected plasma from patients with clinically diagnosed MCI at baseline and followed up with them for 5 years at 1-year intervals. We hypothesized that plasma TTR levels may relate to the rate of cognitive decline and could be a predictor of MCI-to-AD conversion.

## Methods

### Participants

Amnestic MCI subjects were recruited from the outpatient clinics of the Taipei Veterans General Hospital, Taichung Veterans General Hospital, and Changhua Christian Hospital in Taiwan during the period from August 2012 to December 2016. Cognitively healthy controls were also recruited for comparison at baseline. All MCI subjects had an informant who could provide reliable histories, and the age at onset needs to be greater than 60 years. All subjects received a standardized evaluation, including a clinical interview, neuropsychological assessments, laboratory tests, and brain magnetic resonance imaging. A diagnosis of MCI was made according to the revised consensus criteria from 2004^18,19^. The cutoff value for the diagnosis of MCI was set at 1.5 standard deviations below the age-adjusted norm for the logical memory test of the Wechsler Memory Scale III^20^. The exclusion criteria were subjects who had other significant neurological diseases that may have affected cognition and subjects who had any known inflammatory conditions or took anti-inflammatory drugs. The disease duration was defined as the period between the initial onset of symptoms reported by the informant and participation in the study. This research project was approved by the institutional review boards at the three hospitals. All methods were performed in accordance with the relevant guidelines and regulations. Informed consent was obtained from all subjects and their informants before study participation.

### Clinical evaluation and procedures

Cognitive function was assessed by standard procedures at baseline and annual follow-ups. Global cognitive and functional status was assessed by the Mini-Mental State Examination (MMSE)^21^ and Clinical Dementia Rating (CDR)^22^. The 12-item memory test, modified 15-item Boston naming test, category verbal fluency test, forward and backward digit span test, and trail making A test were used to assess short-term memory, language, executive function, attention and working memory, and visuospatial functions, respectively. Depression was evaluated by the short form of geriatric depression scale^23^. Longitudinal follow-up was performed in all MCI subjects at one-year intervals for 5 years. At the follow-up visits, the same clinical evaluation procedure was performed, and the conversion to AD was determined at a multidisciplinary consensus meeting according to the clinical criteria for probable AD as described by the National Institute on Aging–Alzheimer’s Association^24^.

### TTR measurements

Freshly drawn venous blood was collected at baseline in tubes containing EDTA, which were then centrifuged, and the samples were stored in polypropylene tubes at -80 °C until biochemical analysis. The plasma TTR levels were measured in duplicate using a noncompetitive enzyme immunoassay (ELISA Assaypro LLC, prealbumin AssayMAX Human ELISA Kit) according to the manufacturer’s instructions at baseline and at the point when MCI patients converted to dementia. All samples from each participant were measured on the same plate to avoid interplate variation. The coefficient of variance for each sample was less than 20%.

### DNA analysis

Genomic DNA was isolated from whole blood using the Gentra Puregene kit according to the manufacturer’s protocol (Qiagen, Hilden, Germany). The presence of the ε2, ε3, and ε4 alleles of the apolipoprotein E (APOE) gene was determined by assessing the genotype of the participants for the SNPs rs429358 and rs7412. The APOE ε4 carrier was defined as having at least one ε4 allele (including ε2/ε4, ε3ε4, and ε4/ε4). The genotyping of rs429358 and rs7412 was performed using the TaqMan genotyping assay (Applied Biosystems, Foster City, CA, USA). The polymerase chain reactions were performed in 96-well microplates with an ABI 7500 real-time PCR machine (Applied Biosystems). Allele discrimination was achieved by detecting fluorescence using System SDS software version 1.2.3 (Applied Biosystems).

### Statistical analysis

First, frequency matching healthy controls (age- and sex-matched) was used to examine the difference in plasma TTR levels with MCI subjects at baseline by a independent two-sample t-test. Second, according to the diagnosis (maintainging MCI or converting to dementia) at the last visit during the longitudinal follow-ups, MCI subjects were divided into stable MCI patients and MCI converters. Chi-square tests and independent two-sample t-tests were used to examine the differences in demographic and neuropsychiatric variables between the two groups when appropriate. Third, a paired sample t-test was used to examine the difference of plasma TTR levels between at baseline and at the point converting to AD in MCI patients. Fourth, receiver operating characteristic (ROC) curves were applied to examine the predictive ability of plasma TTR level to detect MCI-to-AD conversion at three different follow-up periods (12 months, 24 months, and 36 months) and the areas under the ROC curve (AUC) of the three different time points were calculated. Fifth, survival analysis with time to AD conversion as an event variable was calculated with the multivariable Cox proportional hazards models using plasma TTR level as a continuous variable with the enter method with adjustment for other covariates and bootstrapping procedure with 1000 samples to derive adjusted hazard ratios (HRs) and 95% confidence intervals (95% CI). Addtionally, We divided the plasma TTR level of MCI patients into quartiles to examine the between-quartile relationship of MCI-to-AD conversion. We verified the proportional hazards assumption by plotting log minus log survival curves and the time-dependent covariate method^25^. The statistical analyses were performed with SPSS software (version 20.0, IBM, Inc., Armonk, NY, USA). Statistical significance was defined as p < 0.05.

## Results

### Subjects and demographics

In total, 184 MCI subjects (97 males/87 females; mean age = 76.4 ± 6.4 years; mean education = 10.7 ± 4.6 years) and 40 cognitively healthy controls (16 males/24 females; mean age = 74.8 ± 6.3 years; mean education = 12.1 ± 4.3 years) were recruited for the study at baseline. The mean MMSE score of MCI subjects was lower than that of cognitively healthy controls (25.8 ± 2.5 vs. 27.9 ± 1.7, p < 0.001). The percentage of APOE ɛ4 carriers in MCI subjects was 23.4%. During the longitudinal follow-ups, 135 MCI subjects completed at least one follow-up, and the follow-up rate was 73.4%.

### Baseline plasma TTR levels in MCI patients and healthy controls

MCI patients had higher baseline plasma TTR levels compared with control group (367.4 ± 80.6 vs. 324.6 ± 61.4 ug/ml, p < 0.001).

### Longitudinal follow-up for MCI patients

In the stable MCI subjects, the mean follow-up time was 31.2 ± 16.9 months (range 8–71 months). In the MCI converters, the mean period of maintaining MCI status was 24.0 ± 14.7 months (range 9–60 months) with follow-up intervals of 12.5 ± 2.3 months. Age, years of education, disease duration, and depression scale scores were similar between the stable MCI subjects and MCI converters. The percentage of APOE ɛ4 showed a trend of an increase in the MCI converters (p = 0.05). Additionally, most of the cognitive tests of the MCI converters were worse than those of the stable MCI patients at baseline, including the MMSE (p = 0.003), delayed recall of 12 items memory test (p = 0.03), forward digit span test (p = 0.02), category verbal fluency (p = 0.002), and trail making A (p = 0.001). The plasma TTR levels were similar between the two groups and were also similar between males and females. The detailed demographic and neuropsychiatric data are shown in Table 1.

**Table 1:** Demographic and neuropsychiatric data of MCI patients.

	Stable MCI (n = 73)	MCI converter (n = 62)	*p* value*
Male	43 (58.9%)	30 (48.4%)	0.29
Age	75.6 ± 6.6	77.4 ± 6.2	0.11
Education (years)	11.3 ± 4.2	10.8 ± 4.9	0.55
Disease duration (months)	26.8 ± 26.5	30.6 ± 29.0	0.42
Body mass index	24.6 ± 3.5	23.9 ± 3.7	0.33
Baseline MMSE	26.6 ± 2.2	25.3 ± 2.8	0.003
Delayed recall	5.0 ± 2.4	4.1 ± 2.8	0.03
Forward digit span	10.3 ± 2.2	9.3 ± 2.6	0.02
Backward digit span	5.9 ± 2.1	5.2 ± 2.0	0.06
Category verbal fluency	10.5 ± 3.1	9.0 ± 2.4	0.002
Geriatric depression scale	4.1 ± 3.3	4.0 ± 3.3	0.1
Modified Boston Naming	13.7 ± 1.2	13.4 ± 1.2	0.18
Trail making A (seconds)	74.7 ± 32.7	105.4 ± 65.3	0.001
Plasma TTR level (μg/ml)	360.2 ± 81.9	371.0 ± 80.4	0.44
APOE ɛ4 carrier	12 (16.4%)	20 (32.3%)	0.05

The values indicate means with standard deviations unless otherwise indicated.

*Chi-square test or *t*-test.

### The longitudinal changes of plasma TTR levels of MCI converters

In MCI converters (n = 62), the plasma TTR levels were checked again for 51 patients (82.2%) at the point when they converted to dementia. The plasma TTR level at the point when they converted to dementia was significantly lower than that at baseline (328.6 ± 66.5 vs. 381.9 ± 77.6 ug/ml, p < 0.001).

### ROC curves analysis in different follow-up periods

The AUCs at the 12-month, 24-month, and 36-month for baseline plasma TTR level to detect MCI-to-AD conversion were 0.603, 0.611, and 0.544, respectively.

### Survival analysis of conversion from MCI to AD

**Figure 1:**
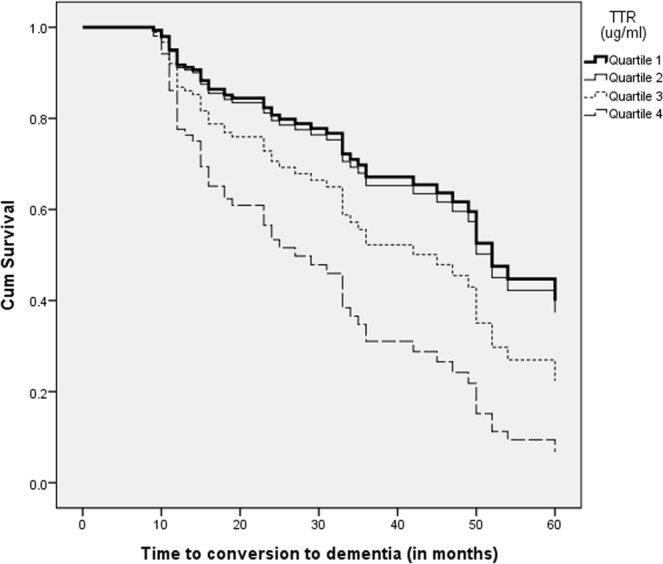
Kaplan-Meier curve for freedom from MCI conversions to AD according to plasma TTR quartiles. There is a dose-response relationship between TTR quartiles and the incidence of AD conversion. The relative risk between extreme quartiles was 2.9 (95% confidence interval = 1.40 to 6.14).

Table 2 showed the Cox model including bootstrapping resampling analysis examining the association between plasma TTR levels and MCI conversion to AD with adjustment for age, sex, years of education, baseline MMSE scores, and APOE ɛ4 carrier status. In the original analysis, the plasma TTR level was an independent factor for the MCI conversion to AD (p = 0.023, 95% CI 1.001–1.007), as well as age (p = 0.006, 95% CI 1.021–1.136), sex (p = 0.036, 95% CI 1.043–3.429), baseline MMSE scores (p = 0.002, 95% CI 0.762–0.939), and APOE ɛ4 carrier status (p = 0.001, 95% CI 1.560–5.545). The results were similar in the bootstrap analysis. This relationship was also evident in the Kaplan-Meier curve for freedom from MCI conversions to AD according to plasma TTR quartiles (Fig. 1) showing a relationship between TTR quartiles and the incidence of AD conversion. The relative risk between extreme quartiles was 2.9 (95% confidence interval = 1.40 to 6.14).

**Table 2:** Results of multivariable Cox regression model.

	Original analysis		Bootstrap analysis	
	HR (95% CI)	*p* value	HR (95% CI)	*p* value
Age	1.077 (1.021, 1.136)	0.006	1.077 (1.025, 1.146)	0.007
Sex				
Female	1.892 (1.043, 3.429)	0.036	1.892 (1.093, 3.615)	0.031
Male	Reference		Reference	
Education	1.019 (0.950, 1.092)	0.601	1.019 (0.944, 1.096)	0.566
Baseline MMSE	0.846 (0.762, 0.939)	0.002	0.846 (0.769, 0.939)	0.001
APOE ɛ4				
Carrier	2.941 (1.560, 5.545)	0.001	2.941 (1.624, 6.123)	0.001
Non-carrier	Reference		Reference	
Plasma TTR	1.004 (1.001, 1.007)	0.023	1.004 (1.000, 1.008)	0.023

HR, hazard ratio; CI, confidence interval; MMSE, Mini-Mental State Examination; APOE, apolipoprotein E; TTR, transthyretin.

## Discussion

The present study demonstrates that MCI patients have higher plasma TTR levels compared with cognitively healthy controls and that higher plasma TTR levels are associated with MCI-to-dementia conversion. Additionally, older age, female sex, baseline MMSE scores, and APOE ɛ4 carrier status are other important factors associated with the conversion from MCI to AD. Furthermore, our results showed decreased plasma TTR levels at the point when MCI patients converted to dementia.

Previous studies have shown that plasma and CSF TTR levels were decreased in both MCI and AD patients as well as AD patients with rapid cognitive decline and severe cognitive impairment^15–17,26^. A study found a difference in TTR levels based on sex, which showed that women had significantly lower plasma TTR levels than MCI and AD men^16^. However, in this study, we did not find any difference in TTR levels between sexes, which is similar to another study^17^. To our knowledge, this is the first longitudinal study investigating the association between TTR levels and the conversion from MCI to AD. To summarize the results from all of the relevant studies, the TTR levels might change dynamically according to the different disease stages. The TTR levels are low in cognitively healthy elderly initially. During the MCI stage, the TTR levels keep increasing and the patients with higher TTR levels tend to convert to AD, but after conversion to the dementia stage, the TTR levels drop and lower TTR levels reflect rapid cognitive decline.

TTR has been reported to be linked to AD pathogenesis because it is able to sequester Aβ40 and Aβ42 and suppress Aβ fibrillation^27^. According to the model of the dynamic AD biomarkers^28^, Aβ accumulation should be an early step in the pathophysiological processes. One possible reason for the increased plasma TTR associated with the conversion from MCI to AD is upregulated TTR levels in the reactive response to the Aβ accumulation during the early stage of AD^29,30^. Higher plasma TTR levels might reflect more rapid or larger amounts of Aβ accumulation, which might result in more rapid cognitive decline with time. The other reason is that TTR levels are associated with dysfunction of the blood-brain barrier (BBB). Some studies have reported the correspondence between the CSF TTR and serum/plasma TTR levels, suggesting that the TTR was permeable through the blood-brain barrier^31^. Another study showed that TTR interacts directly with Aβ at the BBB, stimulates brain-to-blood Aβ permeability, and transports Aβ from but not into the brain^32^. Because BBB breakdown has been shown to be an early biomarker of human cognitive dysfunction^33^ and blood TTR levels could be a possible marker of blood-to-CSF barrier disruption^31^, the dynamic changes of plasma TTR, a transporter protein associated with the permeability of BBB, may reflect the dysfunction of BBB in AD during the different disease stages.

There are many studies analyzing significant biomarkers associated with MCI-to-AD conversion, but the results have been inconsistent. Some studies have demonstrated that different biomarkers, including hippocampal volume, the CSF tau/Aβ-42 ratio, tau proteins and Aβ levels, which had great sensitivity up to 88.9% and specificity up to 96.8%, and the combined assessment outperformed single biomarker assessments^34,35^. Other studies have also shown that absent APOE4 alleles may predict MCI stability for at least 3 years^36^, plasma homocysteine and serum brain-derived neurotrophic factor may predict MCI-to-AD conversion in patients with the APOE ε4 genotype^37^, and plasma beta-secretase 1 activity is significantly increased in MCI converters^38^. Some neuroimaging approaches have provided other ways to predict MCI-to-AD conversion^39,40^. However, MCI is a diverse group, and the progression from MCI to AD is dynamic. Therefore, more studies are needed to investigate the temporal relationship between new biomarkers and clinical disease processes. In the present study, we analyzed the temporal relationship between plasma TTR levels and the conversion from MCI to AD, which provided evidence that plasma TTR is involved in the disease course of MCI-to-AD conversion.

Our study has some limitations. First, the diagnoses of MCI and AD were made by clinical criteria without biomarker evidence of Aβ and tau, which may have influenced the diagnostic accuracy because the MCI patients could have mixed etiology. Second, we did not include other confounding factors that potentially affect TTR levels, such as genetic background, thyroid hormone, and plasma Aβ. Third, some MCI patients might die or convert to AD between two clinical visits, which could confound the results.

## Conclusion

Our study supported the temporal relationship between the plasma TTR level and the conversion from MCI to AD, which may help clinicians predict the prognosis of MCI subjects. Identifying the MCI subjects who have a higher probability of converting to AD may aid clinicians in treating earlier and preventing deterioration. Further studies on the detailed mechanism of TTR in different AD stages should be conducted in the future.

### Ethics approval and consent to participate

This research was approved by the local ethics committees at Taipei Veterans General Hospital (IRB number 2012–05–033B), Taichung Veterans General Hospital (IRB number SF12171), and Changhua Christian Hospital (IRB number 120511). Informed consent was obtained from all patients and their caregivers before study participation.

### Consent for publication

All the authors gave their approval for publication.

## Data Availability

The datasets used and/or analyzed during the current study are available from the corresponding author on reasonable request.

## References

[1] Prince M (2013). The global prevalence of dementia: a systematic review and metaanalysis. Alzheimer’s & dementia: the journal of the Alzheimer’s Association.

[2] Petersen RC (1999). Mild cognitive impairment: clinical characterization and outcome. Archives of neurology.

[3] Grundman M (2004). Mild cognitive impairment can be distinguished from Alzheimer disease and normal aging for clinical trials. Archives of neurology.

[4] Martinez-Torteya A (2018). Identification and Temporal Characterization of Features Associated with the Conversion from Mild Cognitive Impairment to Alzheimer’s Disease. Current Alzheimer research.

[5] Gainotti G, Quaranta D, Vita MG, Marra C (2014). Neuropsychological predictors of conversion from mild cognitive impairment to Alzheimer’s disease. Journal of Alzheimer’s disease: JAD.

[6] Kondo D (2016). Characteristics of mild cognitive impairment tending to convert into Alzheimer’s disease or dementia with Lewy bodies: A follow-up study in a memory clinic. Journal of the neurological sciences.

[7] Maccioni RB, Lavados M, Maccioni CB, Mendoza-Naranjo A (2004). Biological markers of Alzheimer’s disease and mild cognitive impairment. Current Alzheimer research.

[8] Schreiber, G., Richardson, S. J. & Prapunpoj, P. Structure and expression of the transthyretin gene in the choroid plexus: a model for the study of the mechanism of evolution. *Microscopy research and technique* **52**, 21-30, 10.1002/1097-0029(20010101)52:1<21::aid-jemt4>3.0.co;2-z (2001). 10.1002/1097-0029(20010101)52:1<21::AID-JEMT4>3.0.CO;2-Z 11135445

[9] Schreiber G (1990). Thyroxine transport from blood to brain via transthyretin synthesis in choroid plexus. The American journal of physiology.

[10] Schreiber G (1993). Transthyretin expression evolved more recently in liver than in brain. Comparative biochemistry and physiology. B, Comparative biochemistry.

[11] Soprano DR, Herbert J, Soprano KJ, Schon EA, Goodman DS (1985). Demonstration of transthyretin mRNA in the brain and other extrahepatic tissues in the rat. The Journal of biological chemistry.

[12] Wu ZL (2006). Comparative analysis of cortical gene expression in mouse models of Alzheimer’s disease. Neurobiology of aging.

[13] Li X, Masliah E, Reixach N, Buxbaum JN (2011). Neuronal production of transthyretin in human and murine Alzheimer’s disease: is it protective?. The Journal of neuroscience: the official journal of the Society for Neuroscience.

[14] Costa R, Goncalves A, Saraiva MJ, Cardoso I (2008). Transthyretin binding to A-Beta peptide–impact on A-Beta fibrillogenesis and toxicity. FEBS letters.

[15] Velayudhan L (2012). Plasma transthyretin as a candidate marker for Alzheimer’s disease. Journal of Alzheimer’s disease: JAD.

[16] Ribeiro CA (2012). Transthyretin decrease in plasma of MCI and AD patients: investigation of mechanisms for disease modulation. Current Alzheimer research.

[17] Han SH (2011). Human serum transthyretin levels correlate inversely with Alzheimer’s disease. Journal of Alzheimer’s disease: JAD.

[18] Petersen RC (2004). Mild cognitive impairment as a diagnostic entity. Journal of internal medicine.

[19] Winblad B (2004). Mild cognitive impairment–beyond controversies, towards a consensus: report of the International Working Group on Mild Cognitive Impairment. Journal of internal medicine.

[20] Wechsler D. Wechsler Adult Intelligence Scale. 3rd ed. San Antonio, TX: The Psychological Corporation; 1997.

[21] Folstein MF, Folstein SE, McHugh PR (1975). Mini-mental state”. A practical method for grading the cognitive state of patients for the clinician. Journal of psychiatric research.

[22] Morris JC (1993). The Clinical Dementia Rating (CDR): current version and scoring rules. Neurology.

[23] Sheikh JI (1986). 9/Geriatric Depression Scale (GDS) AU - Yesavage, Jerome A. Clinical Gerontologist.

[24] McKhann GM (2011). The diagnosis of dementia due to Alzheimer’s disease: recommendations from the National Institute on Aging-Alzheimer’s Association workgroups on diagnostic guidelines for Alzheimer’s disease. Alzheimers Dement.

[25] Delgado J, Pereira A, Villamor N, Lopez-Guillermo A, Rozman C (2014). Survival analysis in hematologic malignancies: recommendations for clinicians. Haematologica.

[26] Gloeckner SF (2008). Quantitative analysis of transthyretin, tau and amyloid-beta in patients with dementia. Journal of Alzheimer’s disease: JAD.

[27] Schwarzman AL (1994). Transthyretin sequesters amyloid beta protein and prevents amyloid formation. Proceedings of the National Academy of Sciences of the United States of America.

[28] Jack CR (2013). Tracking pathophysiological processes in Alzheimer’s disease: an updated hypothetical model of dynamic biomarkers. The Lancet. Neurology.

[29] Li X (2013). Mechanisms of transthyretin inhibition of beta-amyloid aggregation *in vitro*. The Journal of neuroscience: the official journal of the Society for Neuroscience.

[30] Mangrolia P, Yang DT, Murphy RM (2016). Transthyretin variants with improved inhibition of beta-amyloid aggregation. Protein engineering, design & selection: PEDS.

[31] Terazaki H (2001). Variant transthyretin in blood circulation can transverse the blood-cerebrospinal barrier: qualitative analyses of transthyretin metabolism in sequential liver transplantation. Transplantation.

[32] Alemi M (2016). Transthyretin participates in beta-amyloid transport from the brain to the liver–involvement of the low-density lipoprotein receptor-related protein 1?. Scientific reports.

[33] Nation DA (2019). Blood-brain barrier breakdown is an early biomarker of human cognitive dysfunction. Nature medicine.

[34] Walhovd KB (2010). Combining MR imaging, positron-emission tomography, and CSF biomarkers in the diagnosis and prognosis of Alzheimer disease. AJNR. American journal of neuroradiology.

[35] Nesteruk M (2016). Combined use of biochemical and volumetric biomarkers to assess the risk of conversion of mild cognitive impairment to Alzheimer’s disease. Folia neuropathologica.

[36] Clem MA (2017). Predictors That a Diagnosis of Mild Cognitive Impairment Will Remain Stable 3 Years Later. Cognitive and behavioral neurology: official journal of the Society for Behavioral and Cognitive Neurology.

[37] Zheng L (2016). Conversion from MCI to AD in patients with the APOE epsilon4 genotype: Prediction by plasma HCY and serum BDNF. Neuroscience letters.

[38] Shen Y (2018). Increased Plasma Beta-Secretase 1 May Predict Conversion to Alzheimer’s Disease Dementia in Individuals With Mild Cognitive Impairment. Biological psychiatry.

[39] Moradi E, Pepe A, Gaser C, Huttunen H, Tohka J (2015). Machine learning framework for early MRI-based Alzheimer’s conversion prediction in MCI subjects. NeuroImage.

[40] Adamson C, Beare R, Ball G, Walterfang M, Seal M (2018). Callosal thickness profiles for prognosticating conversion from mild cognitive impairment to Alzheimer’s disease: A classification approach. Brain and behavior.

